# Expression Dynamics of Innate Immunity in Influenza Virus-Infected Swine

**DOI:** 10.3389/fvets.2017.00048

**Published:** 2017-04-21

**Authors:** María Montoya, Emanuela Foni, Alicia Solórzano, Elisabetta Razzuoli, Massimiliano Baratelli, Dania Bilato, Lorena Córdoba, Maria Angeles Martín del Burgo, Jorge Martinez, Pamela Martinez-Orellana, Chiara Chiapponi, David S. Perlin, Gustavo del Real, Massimo Amadori

**Affiliations:** ^1^Centre de Recerca en Sanitat Animal (CReSA), UAB-IRTA, Universitat Autònoma de Barcelona, Barcelona, Spain; ^2^The Pirbright Institute, Woking, UK; ^3^OIE Reference Laboratory for Swine Influenza, Istituto Zooprofilattico Sperimentale della Lombardia e dell’Emilia-Romagna, Parma, Italy; ^4^Public Health Research Institute and Regional Biocontainment Laboratory, Rutgers, The State University of New Jersey, Newark, NJ, USA; ^5^S.S. Sezione Genova, Istituto Zooprofilattico Sperimentale del Piemonte, Liguria e Valle d’Aosta, Genova, Italy; ^6^Laboratory of Cellular Immunology, Istituto Zooprofilattico Sperimentale della Lombardia e dell’Emilia-Romagna, Brescia, Italy; ^7^Department of Biotechnology, Instituto Nacional de Investigación y Tecnología Agraria y Alimentaria (INIA), Madrid, Spain; ^8^Departament de Sanitat i Anatomia Animals, Universitat Autònoma de Barcelona, Barcelona, Spain

**Keywords:** pig, influenza, virus, innate immunity, cytokines

## Abstract

The current circulating swine influenza virus (IV) subtypes in Europe (H1N1, H1N2, and H3N2) are associated with clinical outbreaks of disease. However, we showed that pigs could be susceptible to other IV strains that are able to cross the species barrier. In this work, we extended our investigations into whether different IV strains able to cross the species barrier might give rise to different innate immune responses that could be associated with pathological lesions. For this purpose, we used the same samples collected in a previous study of ours, in which healthy pigs had been infected with a H3N2 Swine IV and four different H3N8 IV strains circulating in different animal species. Pigs had been clinically inspected and four subjects/group were sacrificed at 3, 6, and 21 days post infection. In the present study, all groups but mock exhibited antibody responses to IV nucleoprotein protein. Pulmonary lesions and high-titered viral replication were observed in pigs infected with the swine-adapted virus. Interestingly, pigs infected with avian and seal H3N8 strains also showed moderate lesions and viral replication, whereas equine and canine IVs did not cause overt pathological signs, and replication was barely detectable. Swine IV infection induced interferon (IFN)-alpha and interleukin-6 responses in bronchoalveolar fluids (BALF) at day 3 post infection, as opposed to the other non-swine-adapted virus strains. However, IFN-alpha responses to the swine-adapted virus were not associated with an increase of the local, constitutive expression of IFN-alpha genes. Remarkably, the Equine strain gave rise to a Serum Amyloid A response in BALF despite little if any replication. Each virus strain could be associated with expression of cytokine genes and/or proteins after infection. These responses were observed well beyond the period of virus replication, suggesting a prolonged homeostatic imbalance of the innate immune system.

## Introduction

The first line of defense after viral infections is based on innate immunity, which has the capacity to respond to pathogens by sensing pathogen-associated molecular patterns (PAMPs) through germline-encoded pattern recognition receptors. This process leads to the production of several cytokines such as type I (α and β) interferons (IFNs). These bind to the type I IFN receptor to signal the induction of hundreds of interferon-stimulated genes in the local uninfected and virus-infected cells, thus creating an antiviral state that suppresses virus infection and serves to promote the onset of adaptive immunity (1, 2). Innate immune responses are crucial not only for blunting viral replication in the first instance but also for orchestrating effective adaptive immune responses. Therefore, a transient induction of cytokines and chemokines is required for efficient antiviral responses. However, overreacting and prolonged immune responses may lead to undesired secondary effects, contributing to immunopathology.

As for influenza virus (IV), the innate immune response may vary between mild and severe infections, and include widely different soluble innate immune inhibitors: sialic acid-based inhibitors, Ca-dependent lectin inhibitors, anti-microbial peptides, complement, natural IgM, IFNs, to name a few (3). Toll-like receptors 3 and 7 (TLR-3 and TLR-7) have been found to play an important role in influenza A virus recognition and initiation of the immune response in human respiratory epithelial cells and plasmacytoid dendritic cells (pDCs) (4). Activation of both TLRs and cytoplasmic receptors leads to a potent type I IFN release and simultaneous pro-inflammatory cytokine expression. Type I IFNs, IFN-γ, and pro-inflammatory cytokines, such as interleukin (IL)-1, IL-6, IL-8, IL-12, and tumor necrosis factor-alpha (TNF-α), have been shown to be upregulated in lung tissue and lung lavage after experimental infection of pigs with Swine IV (5–7).

Three IV subtypes (H1N1, H1N2, and H3N2) are currently circulating in swine herds in Europe (8–10) and have been associated with disease occurrence and gross lesions in swine (11). Also, pigs are susceptible to infection with low pathogenic and highly pathogenic avian IVs (LPAIV and HPAIV, respectively) (12). Most LPAIV subtypes diagnosed in field samples possess a H1 or H3 hemagglutinin usually restricted to birds. HPAIV are able to infect pigs under natural and experimental conditions (12). Besides, it is well known that some IVs are able to infect humans and pigs, as it was the case in the last H1N1 2009 pandemic infection (13, 14). *In vitro*, different IV strains can interact with porcine dendritic cells (DCs) and induce sequential waves of cytokine production that are dependent on time and virus strain (15). This effect could account for the different immune responses generated by IV strains. On the other hand, we have previously examined the potential of H3N8 IV from canine, equine, avian, and seal origin to productively infect pigs. Our results demonstrated that avian and seal viruses replicated substantially and caused detectable lesions in inoculated pigs without previous adaptation (16). However, no studies *in vivo* have addressed whether innate immune responses of pigs to swine-adapted and non-adapted IV strains might be different. The above findings outline the main working hypothesis of our study, which implies that Swine IV strains might give rise to peculiar innate immune responses and time courses thereof in pigs, clearly different from those triggered by other IV strains. In order to answer these questions, samples from pigs infected with a Swine (H3N2) and four different non-swine-adapted H3N8 IV strains circulating in different animal species (dogs, horses, wild aquatic birds, and seals) from our previous study (16) were analyzed and innate immune responses in the respiratory tract were thoroughly investigated.

## Animals and Methods

### Animals

In line with the above operational framework, six groups of pigs (7–8 weeks old, Landrace × Pietrain, free from common pathogens) were housed in separate isolation rooms and adapted to the new environment and stockmen over a 1-week period under veterinary supervision. The animals used in our study were seronegative at that time to Influenza A viruses by competition Elisa (ID Screen^®^ Influenza A Antibody Competition ELISA, ID-Vet, France). Also, they had been always healthy and thrifty before the present study.

### Experimental Infection

The experimental infection of pigs with different IV strains was described in our previous paper (16).

Pigs were infected with the following IV strains:

Group 1: 6 pigs, mock-infected.
Group 2: 12 pigs. A/swine/Spain/54008/2004 (H3N2), a Swine IV strain.
Group 3: 12 pigs. A/equine/OH/1/03 (H3N8), an Equine IV strain.
Group 4: 12 pigs. A/canine/NY/105447/08 (H3N8), a Canine IV strain.
Group 5: 12 pigs. A/American black duck/Maine/44411-532/2008 (H3N8), an Avian IV strain.
Group 6: 12 pigs. A/Harbor Seal/New Hampshire/179629/2011 (H3N8), a Seal IV strain.

At day 0, each pig in groups 2, 3, 4, 5, and 6 was intratracheally infected with 1 ml of virus suspension in PBS, containing 2 × 10^5^ Chicken Embryo Infectious Doses 50% (EID_50_) of the corresponding virus strain. Animals were clinically inspected on a daily basis. Rectal temperature and weight were measured at the same times. Four pigs of each virus-infected group were euthanized at day 3, 6, and 21 post infection (p.i.), respectively. In the mock-infected group, two pigs were euthanized at each of the same times.

### Samplings and Postmortem Examination

Blood serum samples in vacuum tubes were collected from jugular veins at day 0 and just before sacrifice at the aforementioned days. After sacrifice, bronchoalveolar fluids (BALF) were collected by lung lavage with PBS, as described by Busquets et al. (14). Briefly, the right lung of sacrificed pigs was used to perform a bronchoalveolar lavage (BAL) using around 100 ml of PBS, and the left one was sampled for histopathological and virological studies. A complete necropsy was performed. Lung lesions were classified depending on the extent of pneumonia. Mild lesions were recorded when affecting small areas (<2 cm^2^) of cranial or medial lung lobes, moderate lesions when affecting extended areas (2–5 cm^2^) of cranial or medial lobes, and severe lesions when affecting large areas (>5 cm^2^) of cranial, medial, diaphragmatic, and accessory lobes.

### Histopathology

Samples from nasal turbinates, trachea, and lungs were collected, fixed in 10% buffered formalin and processed for histopathology, i.e., they were dehydrated through graded alcohols and embedded in paraffin. The 3-µm thick sections were cut, stained with hematoxylin-eosin and examined in a “blind-fashion” manner. In particular, we aimed to evaluate bronchiolar epithelial changes and peribronchiolar inflammation in large, medium, and small or terminal bronchioles, as well as inflammatory changes in alveoli. In the lung, bronchointerstitial pneumonia intensity was assessed by means of a semi-quantitative lesion score. The pathological scores for tracheal and pulmonary tissues were as follows: 0: no lesions, 1: mild lesions, 2: moderate lesions, 3: severe lesions.

### Virus Detection in BALF

In order to check IV replication in the lower respiratory tract of pigs, BALF samples collected from pigs killed at day 3, 6, and 21 were tested for gene M of the influenza A virus using a quantitative real-time PCR procedure (17). The sensitivity of the test was equal to 10^2^ tissue culture infectious doses 50% (TCID_50_)/25 microliters.

### Serum Antibody

Anti-influenza A virus nucleoprotein (NP) antibody levels were investigated in serum using the ID Screen^®^ Influenza A Antibody Competition ELISA (ID-Vet, France) following the manufacturer’s instructions at days 0, 6, and 21 p.i. as previously described (14). The threshold level was set at <50% of the OD level observed in the control wells without any swine serum. The kit detects antibodies against all Influenza A subtypes and antigenic variants thanks to the use of a monoclonal antibody against a highly conserved epitope of the Influenza A virus NP and it was used with pig serum as previously described (13, 14) to avoid background.

### Clinical Immunology Parameters and Cytokines in BALF

Acute phase proteins (APP) of pigs (Haptoglobin and Serum Amyloid A, SAA) were investigated in BALF samples by commercial colorimetric kits (Haptoglobin kit, Tridelta Development, code TP801. Multispecies SAA ELISA Kit, Tridelta Development, code TP802), according to the manufacturers’ directions.

Interferon-alpha was measured in BALF samples by two different assays. First, a sandwich ELISA with monoclonal antibodies (mAb) F17 and K9 to porcine IFN-alpha1 was used as previously described (18). IFN-alpha was also measured by a cytopathic effect inhibition assay on MDBK cells with vesicular stomatitis virus (19). The test was calibrated with a preparation of porcine recombinant IFN-α1 (PBL Biomedical Laboratories, cat. 17100-1). The units of this preparation are determined with respect to the international reference standard for human leukocyte IFN (Ga-902-530) provided by National Institutes of Health (Bethesda, MD, USA). This assay for porcine IFN-α in serum had been also validated in a previous study (20). Identification of the cytokine was performed by a neutralization assay on MDBK cells of IFN α-positive BALF samples (21), using monoclonal antibody (mAb) G16 to porcine IFN-α1 (Serotec, cat. MCA1935Z). The assay was calibrated with porcine recombinant IFN-α1.

Interleukin-6 and TNF-α were determined in BALF samples by bioassays on 7TD1 and WEHI 164 cells, respectively, as previously described (22, 23). Cytokine concentrations were determined from a standard curve created with reference preparations of human recombinant IL-6 and TNF-α (Pierce Endogen, Rockford, IL, USA).

### Flow Cytometry

Immunophenotyping of BALF cells was carried out by flow cytometry. This was performed using indirect labeling of cells by hybridoma supernatants for CD3 (clone PPT3), CD172a (SWC3) (clone 74-22-15a), and Swine MHC (SLA) II (clone 1F12) (24). The antibody against γδ TcR (clone PGBL22A) was purchased from Euroveterinaria S.A. and the one against CD4 (clone 74-12-4) was purchased from Serotec S.A., both conjugated with R-Phycoerythrin (PE). As for the hybridoma supernatants, the concentration of antibody was empirically calculated after titration of each preparation and the secondary antibodies were either PE or APC-conjugated goat anti-mouse IgG (Jackson ImmunoResearch, Suffolk, UK) used following the manufacturer’s instructions. Each primary antibody was compared with its relevant isotype control in each sample using the same concentrations. Briefly, 2.5 × 10^5^ cells/50 μl/well were labeled for 1 h at 4°C with 50 µl of each monoclonal antibody (relevant and irrelevant) at a pre-established, optimal dilution. After 1-h incubation at 4°C, cells were washed with cold PBS with 2% FCS by centrifugation at 450 × g, 4°C, 5 min. Then, the secondary antibody conjugated to R-Phycoerythrin diluted 1:200 was added. Cells were incubated for a further 1 h at 4°C, then washed as before and resuspended in PBS with 2% FCS. Stained cells were acquired using FACSaria I (Becton Dickinson^®^) and analyzed by software FACSDiva v.6.1.2. A gating strategy was applied to living cells using their forward and side scatter (FS/SS) characteristics. Staining with isotype controls did not exceed 1% in all the samples.

### Quantitative Real-time PCR Assays for Cytokine Genes

Total RNA was extracted from BALF cells using RNeasy Mini Kit (Qiagen, Milan, Italy) by the Qiacube System (Qiagen, Milan, Italy) in accordance with the manufacturer’s instructions. The protocol included a DNAse treatment for eliminating genomic DNA. RNA concentration was evaluated by UV absorbance (Biophotometer, Eppendorf, Milan). Three µl of RNA (10 ng/µl) were added to the reaction mix for cDNA synthesis. This was performed in the presence of Random Hexamers as previously described (25). Then, the expression of porcine IFN-α, IL-8, IL-6, IL-1β, and TNF-α genes was determined using the primer sets shown in Table 1. The choice of the porcine IFN-α subtypes shown in Table 1 was dictated by their important role in constitutive expression of type I IFNs (26) and, therefore, by their possible expression before IV infection. Porcine beta2-microglobulin was used as housekeeping control gene (Table 1) to normalize the test results at different sampling times. EvaGreen real-time PCR amplification was performed over 40 cycles in a CFX96 real-time system (Bio-Rad, Milan, Italy) as described by Razzuoli et al. (27). In each sample, the relative expression of the selected genes was calculated using the formula ΔCt = Ct (target gene)—cycle threshold (Ct) (housekeeping), where Ct (cycle of threshold) values were the mean of three test replicates ±1 SD, as previously described (28). Negative samples were given a Ct 40 fictitious value for further statistical examination.

**Table 1:** **Oligonucleotide primer sequences for EvaGreen qRT real-time PCR amplification of porcine genes**.

Gene	Protein	Primers	GeneBank accession number
Interleukin (IL)-8	Porcine IL-8	F: 5′-TTCGATGCCAGTGCATAAATA-3′	AB057440.1
		R: 5′-CTGTACAACCTTCTGCACCCA-3′	
IL-10	Porcine IL-10	F: 5′-AGCCAGCATTAAGTCTGAGAA-3′	AM295341
		R: 5′-CCTCTCTTGGAGCTTGCTAA-3′	
IL-1β	Porcine IL-1β	F: 5′-AATTCGAGTCTGCCCTGTACCC-3′	NM_001005149
		R: 5′-GCCAAGATATAACCGACTTCACCA	
Tumor necrosis factor (TNF)-α	Porcine TNF-α	F: 5′-TGCCTACTGCACTTCGAGGTTATC-3′	NM_214022.1
		R: 5′-GTGGGCGACGGGCTTATCTG-3′	
IFNA5/6	Porcine interferon (IFN)-α5/6	F: 5′-GCACAAATGAGGAGAATATCT-3′	DQ872658.1
		R: 5-CCTCCTGAGTCTGTCTTG-3	
IFNA7/11	Porcine IFN-α7/11	F: 5′-GGGACTTTGGATCCCCTCAT-3′	GQ415065.1
		R: 5′-GTGGAGGAAGAGAAGGATG-3′	
IFNA9	Porcine IFN-α9	F: 5′-GTGCTGCTCAGCTGCAAG-3′	GQ415063.1
		R: 5′-AGTCCTCCTCCAGCAGGGGC-3′	
IFNA13	Porcine IFN-α13	F: 5′-ATCCTCAGCCCTCCTCAC-3′	NM_001164843.1
		R: 5′-ATCCAAAGTCCCTTCTGT-3′	
IFNA16	Porcine IFN-α16	F: 5′-GTTCAGACCCACAGCCTG-3′	NM_001164855.1
		R: 5′-TCCTCCTGAGTCTGTCTTGC-3′	
Beta2-microglobulin	*Sus scrofa*, beta-2-microglobulin	F: 5′-CGCCCCAGATTGAAATTGATTTGC-3′	AB436775.1
		R: 5′-GCTATACTGATCCACAGCGTTAGG-3′	
IFNB	Porcine IFN-β	F: 5-AGTTGCCTGGGACTCCTCAA-3′	JN391525.1
		R: 5-CCTCAGGGACCTCAAAGTTCAT-3′	

### Statistical Analyses

Gene expression data (ΔCt values) obtained on BALF cells were checked for normality by the Kolmogorov–Smirnov test. The data sets passing the normality test were checked for significant differences between time points (days 3, 6, 21 p.i.) by one-way ANOVA for unpaired data, whereas the others were evaluated by the non-parametric Kruskal–Wallis procedure (GraphPad Prism 5.03, GraphPadSoftware, San Diego, CA, USA). The threshold for significance was set at *P* < 0.05.

Statistical analyses on flow cytometry data were carried out using the SAS system V.9.1.3 (SAS institute Inc., Cary, NC, USA). For all analyses, the individual pig was used as the experimental unit. Shapiro Wilk’s and Levene tests were used to evaluate the normality of the distribution of the continuous variable and the homogeneity of variances, respectively. A statistical analysis was performed to study the association between the different variables with the different experimental groups at the different time points (0, 3, and 21 days post-infection). To analyze the association between continuous, normally distributed variables and the different experimental groups, an ANOVA test was used at 0, 3, and 21 days post-infection. Finally, a Wilcoxon rank-sum test was used for the continuous, non-normally distributed variables at the different time points.

## Results

### Clinical and Postmortem Findings

No clinical signs were shown during the experimental procedure, such as fever, weight loss, anorexia, depression, nasal discharge, or altered breathing. Temperature was not higher than 40.5°C in any animal at any time point. There were no traumatic lesions as a result of intratracheal virus inoculation. Gross lesions were mostly shown by animals infected with the swine IV, but some lesions were also detected in lungs from pigs infected with other IV strains. Pulmonary lesions (Table 2) were consistent with bronchointerstitial pneumonia at different grades of severity (0–3). The pigs infected with Swine IV presented the highest level of severity followed by those infected with Seal IV, Avian IV, and either Equine or Canine IV in descending order.

**Table 2:** **Macroscopic lesions observed in controls and pigs inoculated with different IVs**.

Days p.i.	Mock	Swine IV	Equine IV	Canine IV	Avian IV	Seal IV
3	0 (0)	2.5 (0.6)	0.3 (0.5)	0.3 (0.5)	1.0 (0.8)	1.5 (1)
6	0 (0)	2.0 (0.8)	0.5 (0.6)	0.3 (0.5)	1.0 (0.5)	1.3 (1.3)
21	0 (0)	1.0 (0.8)	0.5 (0.6)	0.5 (0.6)	0.5 (0.6)	0.5 (0.6)

*Macroscopic lesions observed in mock and IV-infected pigs. Gross lesions were classified depending on the extension of pneumonia (0–3). Data represent the average for each group; SD is shown in brackets*.

*0 = absence of lesions, 1 = mild lesions, 2 = moderate lesions, 3 = severe lesions*.

### Histopathology

**Figure 1:**
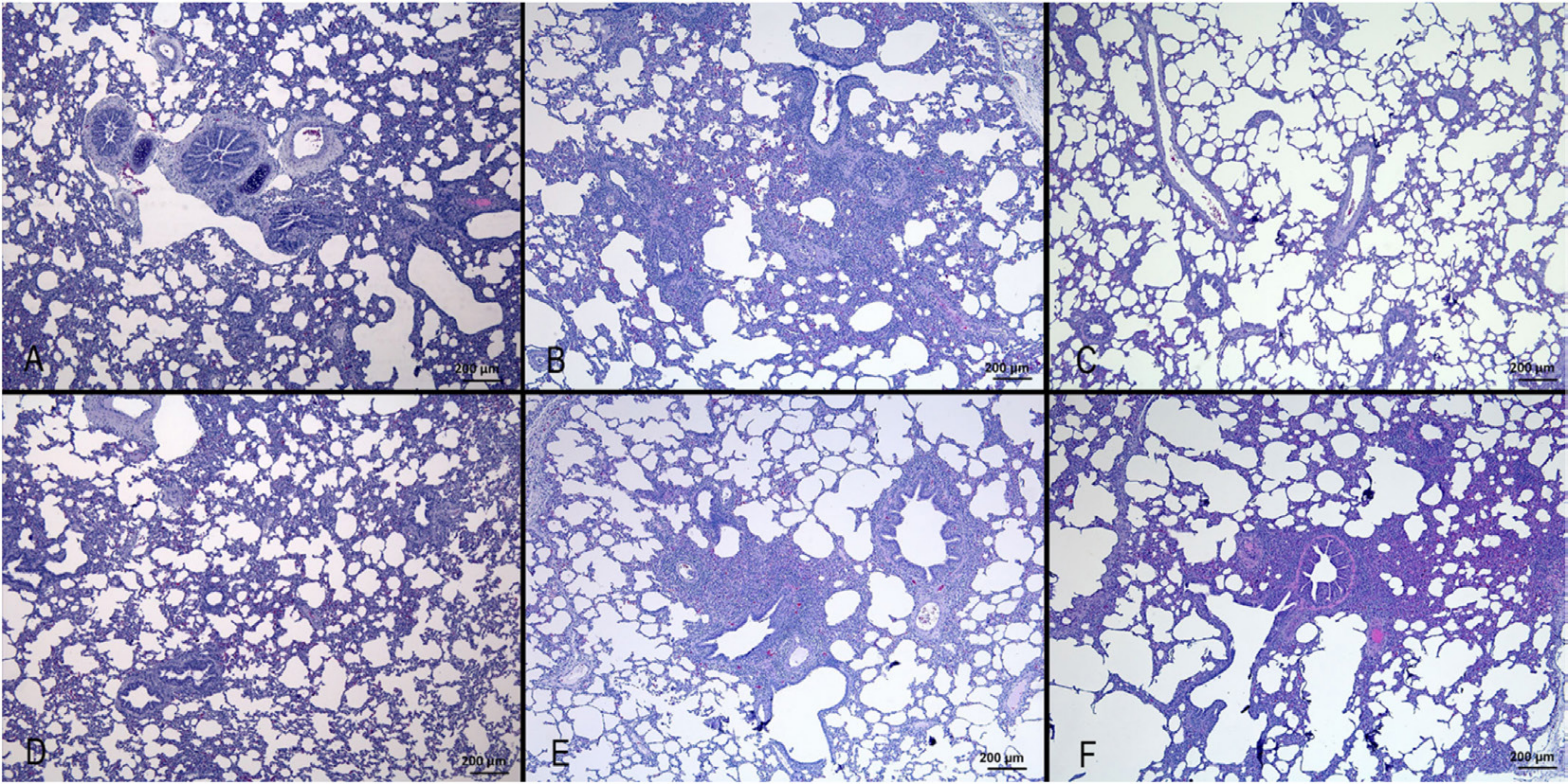
**Histopathological analysis of lungs of pigs, at day 3 after infection with different IVs**. **(A)** Negative control; **(B)** swine IV, moderate to severe inflammation of the bronchioli and surrounding alveoli (bronchointerstitial pneumonia); **(C)** equine IV, no significant lesions; **(D)** canine IV, no significant lesions; **(E)** avian IV, moderate bronchointerstitial pneumonia; **(F)** seal IV, mild to moderate bronchointerstitial pneumonia.

Results are shown in Figure 1. Nasal turbinates sections did not differ between IV and mock-infected animals. Tracheal lesions of IV-infected pigs consisted of variable grades (0–3) of lymphoplasmacytic infiltration in the lamina propria. In the most severe cases, focal ulceration of the mucosa with fibrin deposition and neutrophilic infiltration was observed. A lymphoplasmacytic infiltration was observed in the bronchioli with extension to surrounding alveolar septa. In the most severe cases, at day 3 p.i., necrosis of the bronchiolar epithelium was observed with accumulation of necrotic cells in the bronchiolar lumen and alveolar spaces. In agreement with the gross lesion scores shown in Table 2, histopathological lesions were much more severe in pigs of the Swine group at day 3 p.i. followed by the animals infected with Seal or Avian IV. The pigs infected with either Equine or Canine IV showed the least severe lesions (Table 3).

**Table 3:** **Histopathological analyses of tracheal and lung tissues from mock and IV-infected pigs**.

Days p.i.	Mock		Swine IV		Equine IV		Canine IV		Avian IV		Seal IV	
	T	L	T	L	T	L	T	L	T	L	T	L
3	0 (0)	0 (0)	1.5 (1)	2.3 (0.5)	0.3 (0.5)	0.5 (0.6)	0.3 (0.5)	0 (0)	1.3 (0)	2 (0.8)	1 (1.2)	1.8 (1.5)
6	NP	NP	1.5 (0.5)	0.3 (1.3)	NP	NP	NP	NP	1.3 (0.5)	0.5 (0.6)	1.5 (1.3)	0.5 (1)
21	NP	NP	0 (0)	0 (0)	NP	NP	NP	NP	0.8 (0.5)	0.8 (0.8)	0.3 (0.5)	1.3 (0.6)

*T, trachea; L, lung, NP, not performed*.

*For each tissue, the pathological score varied from 0 to 3; data represent the average for each group (4 and 2 animals in IV and mock groups, respectively); the SD is shown in brackets*.

### IV Infection

**Figure 2:**
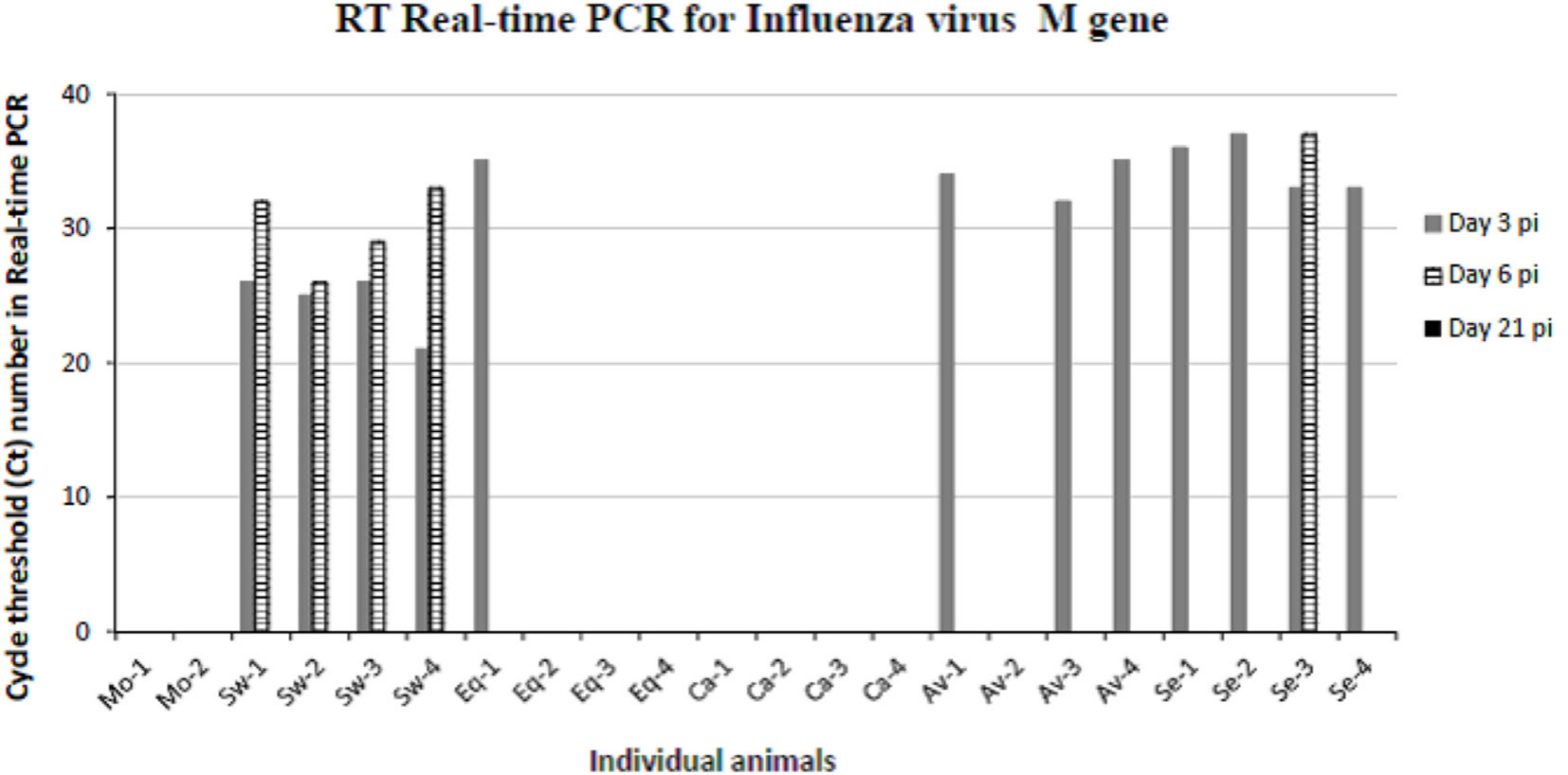
**Results obtained by RT real-time PCR for IV M gene on bronchoalveolar fluids samples collected from mock and IV-infected pigs**. Positive samples are shown as cycle threshold number in real-time PCR. PCR-negative samples corresponded to Ct ≥ 40, and they are not shown in the graph. Two and four pigs were present in each mock and IV-infected group, respectively. Samples from day 3 p.i. are shown as gray bars and from day 6 p.i. as striped bars. All day 21 samples (black bars in the legend) were PCR-negative.

To confirm the virological data (IV titration in embryonated chicken eggs) shown in our previous paper (16), viral replication was evaluated by quantitative real-time PCR on BALF samples of animals sacrificed at days 3, 6, and 21 p.i. (Figure 2). The swine-adapted IV actively replicated in the pig respiratory tract at both day 3 and 6 p.i., as shown by Ct values ranging from 21 to 33. Instead, we could detect only one IV-positive BALF sample in the Equine group at day 3 p.i. with a Ct value of 35 and none in the Canine group, indicating very little replication of those viruses. On the contrary, the Avian and Seal virus strains showed a moderate replication at day 3 in most pigs, with Ct values ranging from 33 to 37. One animal from the Seal group was still IV-positive at day 6 p.i. with a Ct value of 37. The above PCR data are in agreement with IV titrations in embryonated chicken eggs, reported in our previous paper (16).

### Antibody Response

**Figure 3:**
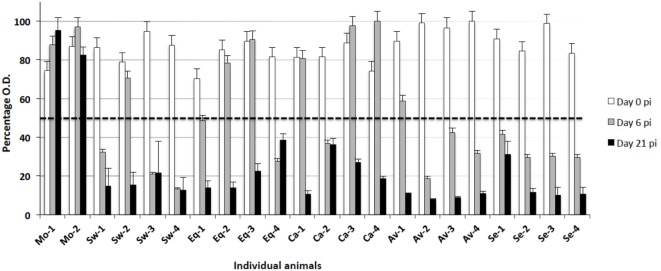
**Antibody responses to different IV strains were investigated by means of a competition ELISA for the nucleoprotein antigen**. The *Y* axis indicates the percentage of OD with respect to the control reaction without any serum (100%). The horizontal dashed line corresponds to the test threshold, i.e., 50% of the control OD value. Ab-positive sera show percentage values beneath the threshold. The *X* axis indicates the pigs under study at three sampling days p.i. (0, 6, 21). Mo, mock-infected; Sw, swine IV-infected; Eq, equine IV-infected; Ca, canine IV-infected; Av, avian IV-infected; Se, seal IV-infected. Samples from day 0 are shown as white bars, from day 6 p.i. as gray bars, and from day 21 p.i. as black bars.

Total anti-NP antibody levels were measured by a competition ELISA kit in serum samples from all animals at the beginning of the assay (day 0), days 6, and 21 p.i. All animals were seronegative at the beginning of the experiment (day 0, Figure 3). Also, no antibody response was observed in mock-infected animals. On the other hand, all IV-infected animals seroconverted (OD < the threshold) by day 21 p.i. Most of them seroconverted by day 6 p.i. in groups infected with Swine, Avian, and Seal viruses, whereas the response was delayed in groups infected with the Equine and Canine viruses (Figure 3).

### Innate Immune Responses

The above findings confirmed a different invasiveness in pigs of swine-adapted and non-adapted IV strains in terms of virus replication and pathological lesions. On this basis, we investigated innate immune responses during IV infection in the respiratory tract that might differ between pathogenic and non-pathogenic IV strains in pigs.

**Figure 4:**
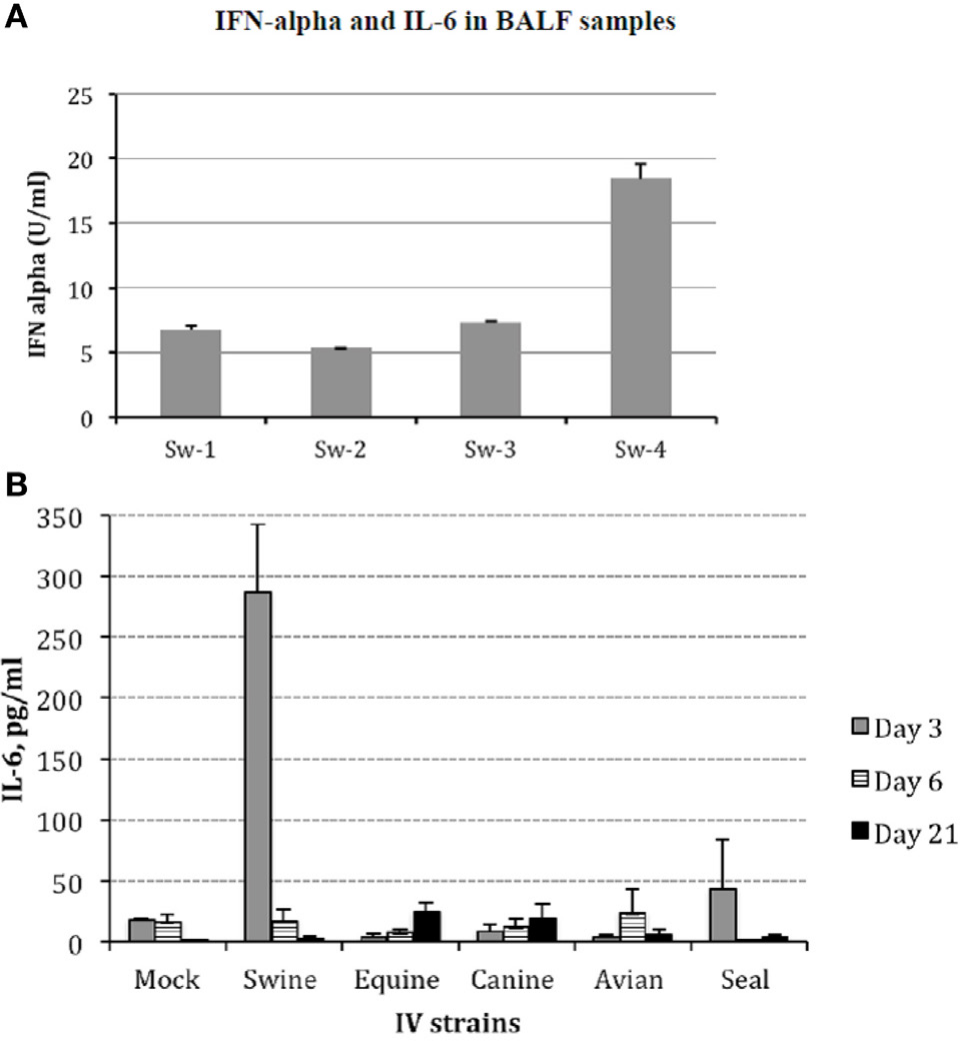
**(A)** Interferon (IFN)-α was investigated in bronchoalveolar fluids (BALF) samples of Swine IV-infected pigs at day 3 p.i. by sandwich ELISA with monoclonal antibodies F17 and K9 to porcine IFN-α1. Results are shown in terms of Units/ml (mean + 1 SD) on the basis of a standard curve created with porcine recombinant IFN-α1. IFN-α titers <1 U/ml were the baseline in our assays. The BALF samples of mock and all other IV groups were always IFN α-negative at all the time points. **(B)** Interleukin (IL)-6 was measured in BALF samples by a bioassay on IL 6-dependent 7TD1 hybridoma cells. On the basis of both final number and viability of 7TD1 cells, IL-6 concentration was determined from a standard curve created with a reference preparation of human recombinant IL-6 (Pierce Endogen, Rockford, IL, USA). Results are shown in terms of group average +1 SEM. The IL-6 concentration in the swine group at day 3 p.i. is significantly different (one-way ANOVA, *P* < 0.05). Please notice that the result of the Seal group at day 3 p.i. was accounted for by an outlier and three non-responder pigs. Samples from day 3 p.i. are shown as gray bars, from day 6 p.i. as striped bars, and from day 21 p.i. as black bars.

Protein cytokine levels were measured in all animals from all groups by different assays. Firstly, levels of IFN-α in BALF were analyzed by ELISA. All samples were negative except those collected from animals infected with Swine IV at day 3 p.i. (Figure 4A). These findings were confirmed by a bioassay for type I IFN on MDBK cells, and the specificity of the response was confirmed by an IFN-neutralization assay based on a mAb to porcine IFN-alpha 1 (data not shown).

There was only one pig (Sw-2) with a slight TNF-α response in BALF at day 3 p.i. in the Swine IV-infected group. BALF samples were always TNF α-negative in the other groups (data not shown).

On the other hand, IL-6 was clearly detected at day 3 p.i. in the BALF samples of Swine IV-infected pigs. In the other groups, there was no significant difference with respect to mock animals, with the exception of pig Se-4 in the Seal group (day 3 p.i.) (Figure 4B).

The high IL-6 response in BALF of the Swine group at day 3 p.i. led us to investigate haptoglobin and SAA in BALF samples of the Mock, Swine, and Equine groups at day 3 p.i. All the samples were haptoglobin-negative (<0.01 mg/ml). On the contrary, all the Equine IV-infected pigs were SAA-positive (range: 2.9–5.8 mg/ml), as also was one sample of the Swine group (4.7 mg/ml). All the other samples were SAA-negative (<0.01 mg/ml).

### Cytokine Gene Expression Studies

**Figure 5:**
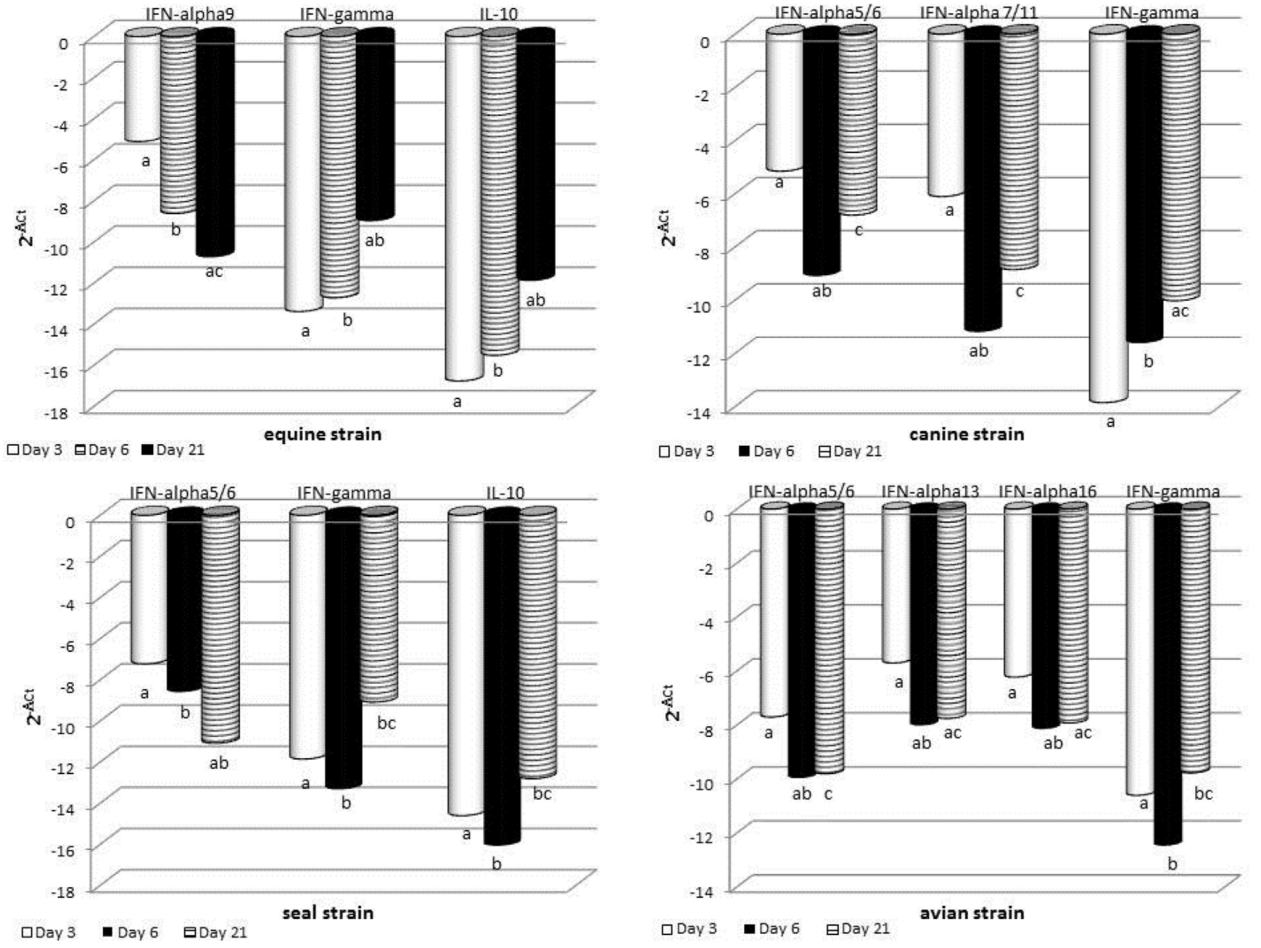
**RT real-time PCR for some cytokine genes was carried out on bronchoalveolar fluids cells collected at the indicated times after infection**. For each sample, the relative expression of the selected genes was calculated using the formula ΔCt = Ct (target gene)—cycle threshold (Ct) (housekeeping), where Ct values were the mean of three test replicates. Results are shown as *n*-fold change in gene expression (2^−ΔCt^). The same superscripts (a, b, c) on the bar indicate significant differences (*P* < 0.05) in one-way ANOVA or Kruskal–Wallis test. Samples from day 3 p.i. are shown as gray bars, from day 6 p.i. as striped bars, and from day 21 p.i. as black bars.

The expression of some cytokine genes was investigated by quantitative real-time PCR on BALF cells. Results are shown in Figure 5 and detailed hereunder. *MOCK group*: there was no significant difference between the different sampling times for each cytokine gene under study. *SWINE group*: there was no significant difference of gene expression at the three time points. Only tendencies (*P* < 0.10) were shown for IL-8, IL-10, and IFN-gamma genes. *EQUINE group*: significant differences were shown for IFNA9, IFN-gamma, and IL-10 genes. In particular, at day 21 p.i., an increase was observed of IFN-gamma and IL-10 and a decrease of IFNA9 gene expression. *CANINE group*: significant differences were shown for IFNA5/6, IFNA7/11, and IFN-gamma genes. At day 6 p.i., there was a downregulation of IFNA5/6 and 7/11, as opposed to a significant increase of IFN-gamma gene expression at day 21 p.i. No other significant change of expression was detected. *AVIAN group*: there were significant differences for IFNA5/6, IFNA13, IFNA16, and IFN-gamma gene expression and tendencies for IFNA7/11 and IFNA9 genes. In particular, a downregulation of these genes occurred at day 6 p.i. *SEAL group*: IFNA5/6 was downregulated at day 21 p.i., whereas IFN-gamma and IL-10 genes were upregulated at the same time.

### Immunophenotyping of Leukocytes

**Figure 6:**
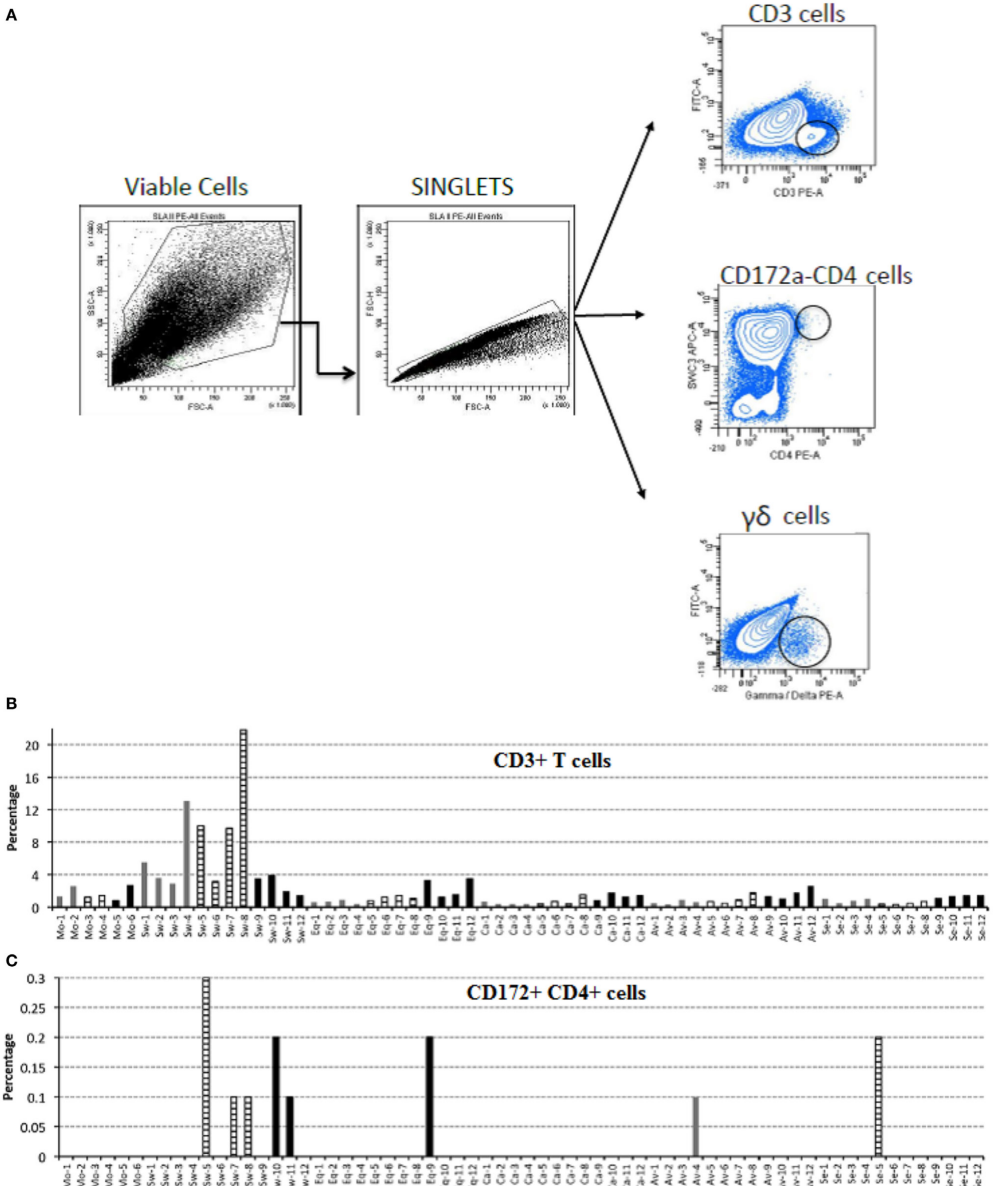

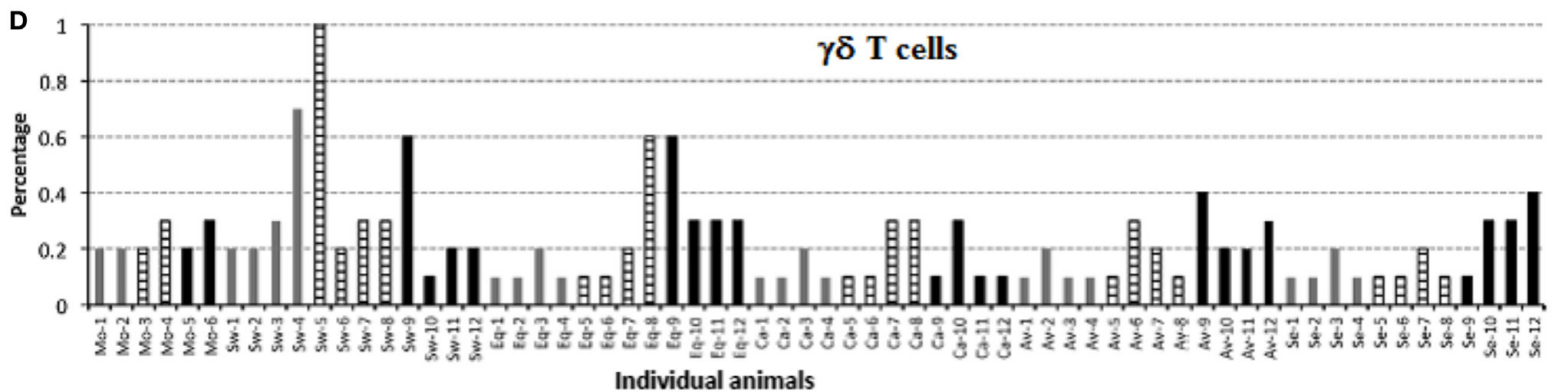
**Immunophenotyping of pig bronchoalveolar fluids (BALF) cells after mock and IV-infection**. BALF cells of pigs were submitted to immunophenotyping at the indicated times after mock or IV infection. Results are expressed in terms of percent marker-positive cells out of 10,000. **(A)** Gating strategy for flow cytometry. Positive cells were considered as the percentage within the black circle in each plot as compared with each isotype control. **(B)** Percentage of CD3-positive T cells. **(C)** Percentage of CD172-CD4, double-positive cells. **(D)** Percentage of γδ T cells. Samples from day 3 p.i. are shown as gray bars, from day 6 p.i. as striped bars, and from day 21 p.i. as black bars.

Based on the fact that local responses in BALF from Swine IV-infected animals were different from those of the other IV-infected pigs, we investigated whether there was any difference in cells infiltrating the lungs of infected pigs (Figure 6). Therefore, cells from BALF were analyzed by flow cytometry to detect changes among animals of each group. Swine IV-infected animals had significantly more T cells (CD3+) at day 3 and 6 p.i. than the rest of the infected groups (*P* < 0.05), and the highest concentrations also corresponded to the appearance of a low concentration of the γδ T cell receptor phenotype. At day 3 p.i., γδ T cell percentages were significantly different from those of the other infected pigs (*P* < 0.05). Also, only Swine IV-infected animals showed a clear increment of pDCs (defined as CD172a + CD4 + cells) at day 6 p.i., with a statistical tendency (*P* = 0.06). No major changes were detected concerning the surface expression of SLA-II or CD172 in BALF cells (data not shown).

## Discussion

In this study, the pathogenicity of IV strains with different host specificity was characterized in swine in terms of clinical symptoms and tissue lesions. Also, we aimed to define a possible association between innate immune responses to IV strains, and clinical and *postmortem* findings.

The main findings of our study are outlined in Table 4. Despite wide variations among individual pigs, the IV strains under study could be discriminated in terms of pathogenicity. Our results confirmed a widely different replication of IV strains in the lower respiratory tract of swine: the swine-adapted virus replicated to a large extent, as opposed to the other IV strains under study. These PCR results were fully in agreement with virus titration results in our previous study (16). Interestingly, the avian and seal H3N8 strains replicated more than the equine and canine strains. Replication of the canine and equine strains could have transiently taken place at days 1–2 post infection, before the first sampling. This is consistent with low-grade postmortem lesions and histopathological analyses on samples at day 3 p. i., as reported in Tables 2 and 3. These findings highlight different invasiveness of Swine, Seal and Avian, Equine, and Canine viruses, in descending order (Figure 2). The replication to a higher level of Swine, Avian, and Seal IV was associated with an earlier antibody response compared with groups Equine and Canine (Figure 3), where a possible low-titered replication could account for the late antibody response to NP. Also, we cannot rule out that defective or partially assembled particles in the viral inoculum could also have induced antibodies to NP, and that different IV strains could elicit antibodies with different reactivity in the assay. Please notice, however, that amino acid identity of NPs with respect to swine-adapted IV was very high, ranging between 92% (canine strain) and 97% (avian and seal strains). Therefore, the combination of an Ab response to IV NP with macro and microscopic lesions and the clear discrimination from mock-infected animals indicate that pigs can be productively infected by H3N8 viruses, which is also in agreement with the replication of both seal and avian H3N8 strain in tracheal explants (16).

**Table 4:** **Main findings of experimental IV infection in pigs**.

IV strains								Modulation of cytokine gene expression in bronchoalveolar fluids (BALF) cells^b^						
	Histopathological lesions	Replication in lungs (PCR)	Ab response	Interferon (IFN)-α in BALF	Interleukin (IL)-6 in BALF	SAA in BALF	BALF: cell infiltrates	IFN A5/6	IFN A7/11	IFN A9	IFN A13	IFN A16	IFN-γ	IL-10
Mock	−	−	−	−	−		−							
Sw	++	++	++	++	++	±	++^a^							
Eq	±	±	+	−	−	++	−			+			+	+
Ca	±	−	+	−	−		−	+	+				+	
Av	+	+	++	−	−		−	+			+	+	+	
Se	+	+	++	−	−		−	+					+	+

*^a^CD3 T cells, γδ T cells, plasmacytoid dendritic cells, as evaluated by flow cytometry*.

*^b^Based on ΔCt values at each time point*.

Most importantly, the pathogenic role of swine-adapted IV strain was confirmed by both gross and histological lesions in lungs, not induced by the other viruses under study. In turn, the presence of lesions could be temporarily associated with local innate immune responses in the lower respiratory tract (BALF samples): IFN-α and IL-6 (Figure 4). These findings are in agreement with those of other experimental infections of pigs with IVs (5, 6). Moreover, previous results in our group showed that IFN-α secretion was only detected *in vitro* when Swine IV-infected myeloid porcine DCs but not when other IVs from human or avian origin were used (15). Interestingly, the IFN-α titers in BAL fluids of swine IV-infected pigs were not associated with the expression of IFN-α genes, in agreement with our previous data *in vitro* (25) and another study *in vivo* (29).

But for one animal, the IL-6 local response in BALF samples of the Swine group at day 3 p.i. was not in agreement with the presence of APP. Unexpectedly, SAA was clearly expressed in BALF samples of the Equine group at day 3 p.i. This indicates that SAA responses can be induced despite poor virus replication in the lower respiratory tract. Also, the Equine strain widely affected the expression of cytokine genes (see Figure 5). This result suggests that innate immune responses can be triggered by some IV strains regardless of their replication efficiency. Also, our findings confirm the possible extrahepatic expression of APP observed in other disease models (30).

The Swine IV strain also caused detectable changes of the BALF-infiltrating leukocyte populations. In particular, some CD4+CD172a+ cells were observed in BALF from Swine IV-infected animals but not in BALF from the other IV groups. These are considered pDCs in swine (31), and their main function is the release of high levels of type I IFNs. This is in agreement with the fact that IFN-α was only observed in BALF samples of the Swine IV-infected group (Figure 4A). IFN-α was detected at day 3 p.i., whereas the increased prevalence of pDCs was detected at day 6 p.i. This apparent discrepancy could be explained by our detection limit for pDCs in BALF, as a certain minimum concentration would probably be required for detection by flow cytometry. Also, we do not rule out a distinct or overlapping release of IFN-α by alveolar macrophages or other cells (e.g., epithelial cells) at an early time point after infection.

Despite the lesions caused by the Swine IV strain, no disease signs were observed. This result is in contrast with an experimental infection of pigs with a Danish H1N2 IV strain, which led to the development of typical clinical signs of influenza (29). The absence of clinical signs was probably associated in our study with the reduced infectious challenge (2 × 10^5^ EID_50_), which was necessary to standardize the challenge infection with IV strains showing high or poor replication in embryonated chicken eggs (16). Yet, our data are in agreement with the fact that many pigs become infected with one or more IV subtypes without showing clinical signs (32).

The gene expression data point at a crucial neglected issue: IV strains seem to cause a long-term regulation of the innate immune response, by far beyond the actual period of virus replication and persistence in organs and tissues. Instead, the modulation of cytokine genes was more related to tissue damages, as indicated by the gross and hystopathological lesions persisting in some pigs until day 21 p.i.

Retrospectively, earlier sampling of lung tissues and/or BALF samples could have provided a more clear picture of cytokine gene expression levels, which were probably in a descending phase at day 3 p.i. after a possible major peak at day 1 (29). This is particularly true of IFN-β, which is highly up-regulated for a short time after IV infection (29). Indeed, our previous results actually showed that IFN-β gene upregulation was only detected 8 h after Swine IV infection of myeloid porcine DCs *in vitro* (15). This is also in agreement with its role as immediate-early type I IFN gene in non-lymphoid cells (33).

Some innate immune responses were triggered by the swine-adapted IV strain, only. Diverse IV strain-specific factors could account for these responses, such as hemagglutinin receptor specificity and affinity, polymerase efficiency and activity of IV anti-IFN proteins, which may show additive or synergistic interactions with the host’s immune system. On the basis of our previous results on tracheal explants of pigs (16), components other than affinity for sialic acid receptors of Swine IV strains are likely to account for high innate immune responses. In particular, gross and histological lesions are probably induced following recognition of PAMPs of the swine-adapted IV strains and activation of the NLRP3 inflammasome (cryopyrin) (34). Such a response is probably less intense after infection with other IV strains, which can be accounted for by different causes: (A) amino acid changes of some viral PAMPs. (B) Suppressive regulations by ODNs of non-swine IV strains [as shown, e.g., for Porcine Circovirus 2 (35), and/or by IFN-α subtypes with anti-inflammatory control activity (36)]. (C) Third, the innate immune response to the swine-adapted IV could be simply due to the much higher and/or quicker replication of this strain. In this respect, viral replication measured by quantitative real-time PCR correlated with the degree of lesions. Beyond the above hypotheses, a biological explanation of the observed results could be obtained by reverse genetics studies: changes or disruptions should be engineered in the viral genome, and effects of such alterations should be checked *in vitro* or *in vivo*.

On the whole, the emerging picture outlines a host/virus relationship characterized by a strict control of the innate immune responses of pigs to non-adapted IV strains. The swine-adapted ones can circumvent these controls, in the framework of a rapid, effective, non Ab-dependent containment of the primary virus infection in the lower respiratory tract. Interestingly, the uncomplicated exposure to the Swine IV strain did not induce clinical signs in our study. This finding points at a crucial role of the infectious pressure caused by common pathogens on farm for disease onset. This feature probably exerts additive and/or synergistic effects with common environmental stressors (animal density, seasonal changes, hierarchy fights after commingling) and genetic traits. As a result, fundamental components of the innate immune response would be affected, and clinically overt influenza outbreaks could take place on farm with full-blown clinical pictures.

## Ethics Statement

All animal experiments were conducted at CreSA, Barcelona, in compliance with the Ethical Committee for Animal Experimentation of the institution (Universitat Autònoma de Barcelona). The treatment, housing, and husbandry conditions conformed to the European Union Guidelines (Directive 2010/63/EU on the protection of animals used for scientific purposes). Animal care and procedures were in accordance with the guidelines of the Good Laboratory Practices (GLP) under the supervision of the Ethical and Animal Welfare Committee of the Universitat Autònoma de Barcelona (number of approval: 1189) and under the supervision of the Ethical and Animal Welfare Committee of the Government of Catalonia (number of approval: 5796).

## Author Contributions

MM supervised the entire study. ER performed real-time PCR assays for cytokine genes and data analysis thereof. MB and PM-O performed flow cytometry experiments and data analysis thereof. DB, LC, and JM supervised sample collection, storage and distribution, as well as cytokine, clinical immunology, histopathology, and antibody assays. EF and CC performed real-time PCR assays for IVs and data analysis thereof. AS, DP, MAB, and GR provided scientific support. MA supervised clinical immunology assays, cytokine protein assays, and the manuscript writing.

## Conflict of Interest Statement

The authors declare that the research was conducted in the absence of any commercial or financial relationships that could be construed as a potential conflict of interest.

## Abbreviations

FCS, fetal calf serum; PCR, polymerase chain reaction; OD, optical density; ODNs, oligodeoxynucleotides; PBS, phosphate buffered saline; RT, reverse transcription; TcR, T cell receptor.
